# Motives for walking and cycling when commuting – differences in local contexts and attitudes

**DOI:** 10.1186/s12544-021-00502-5

**Published:** 2021-08-09

**Authors:** Kristina Ek, Linda Wårell, Linda Andersson

**Affiliations:** Economics Unit, Department of Social Sciences, Technology and Arts, Luleå University of Technology, Luleå, Sweden

**Keywords:** Transport choice, Walking/cycling, Motives, Local context

## Abstract

**Background:**

The purpose of this study is to analyse what factors that explain individual differences in walking and cycling when commuting in different parts of Sweden. Walking and cycling is potentially accessible all over the country, while well developed public transport is mainly a viable option in densely populated areas.

**Methodology:**

The importance of differences in local characteristics for the choice of transport mode will be scrutinised, together with individual differences in attitudes andpreferences. Data is collected through a survey sent to people living in five Swedish municipalities with different demographic, socio-economic ,infrastructural and geographical characteristics.

**Results:**

The results for the pooled sample indicate that the choice to walk/cycle when commuting is related to health considerations and environmental concerns. Distance to work/school is also an important factor. Men tend to be more prone to choose active transport, and so do respondents with lower income. The results further reveal that availability of safe routes for walking and cycling are important for the choice to walk/cycle when commuting. As health considerations are important, we suggest policy makers to stress health motives when they promote walking and cycling in the future. Our results further suggest that it is important to consider availability and accessibility in community planning, and to prioritize safety and comfort of walking and cycling, not least in parts of the country where public transport is not an economically viable option.

## Introduction

In Europe, greenhouse gas emissions from the transport sector in 2017 amounted to 27% of all emissions, and in order to reach the target set in the EU 2011 Transport White Paper, emissions need to decrease with about 70% by 2050. As road transports constitute the major share of these emissions (European Environment Agency, [11]), there is an urgent need for policy makers to understand more about how the society can change to fossil-free transport modes. In a survey conducted by the European Union in 2014 it is found that on a typical day, the most common mode of transport is car (54%), followed by pubilc transport (19%), which is followed by walking (14%) and biking (8%) [10].

Consequently, researchers, policy makers and practitioners are directing massive interest towards reducing the use of non-sustainable alternatives, mainly fossil-fuelled cars, but also to increase the use of public transport ([3, 9, 21, 31], to mention a few). To provide attractive and cost effective public transport for all citizens is however not trivial in a sparsely populated country (like Sweden) with low population density and many small municipalities. Although the flexibility of the transport system may increase in the future, as a result of ongoing digitalisation and technology development, specific challenges (related to e.g. diseconomies of scale and distributional factors) associated with the transformation of the transport system outside metropolitan areas will likely remain (see e.g. [38]).

The transport modes considered most sustainable; i.e. walking and cycling [7], do not emit any hazardous substances or greenhouse gases and they also generate positive health effects [4]. To walk or cycle is an alternative available at relatively low cost also outside metropolitan areas, at least for people in the relative proximity of smaller city centres, and these are accessible travel modes that are available to many travellers.^1^ To increase the use of these active transport modes is an important – but perhaps somewhat under-utilised – way to make the transport system more sustainable and at the same time acquire an improved health status of the population.

The purpose of this study is to examine what factors that explain individual differences in choosing walking or cycling when commuting to work/study. The importance of differences in local characteristics, as well as individual differences in attitudes and preferences will be scrutinised. Data is collected through a survey sent to people living in five Swedish municipalities with different socio-demographic, infrastructural and geographical characteristics.^2^ Data is analysed by applying a binary logit model. The conditions for different travel modes differ across the country, and depend on e.g. population density, density of the city itself, and access to infrastructure. The municipalities included in the analysis are chosen as they vary considerably regarding these characteristics. Differences in individual attitudes towards the environment, climate and health, are also important to understand in order for policy makers to design effective policies that promote more sustainable travel modes over the whole country.

Previous research on factors affecting the choice of travel mode is extensive, but less focus is directed towards walking and cycling. There are studies reporting that factors related both to socio-demographics and the built environment influence the choice to walk and cycle [6, 12, 19, 37]. Plaut [27] analyse non-motorised commuting in the US (walking, cycling or working at home) and finds that individuals with higher income more often work at home, but are less likely to walk or cycle, and that individuals with higher education more often choose non-motorised travel modes. Plaut further finds that variation in the likelihood of using non-motorised travel modes depends both on regional aspects, and on where a person lives within a metropolitan area. An and Chen [2] find that employment density, household income and average sidewalk length affect the choice of walking/cycling. Also, gender, age, car-ownership, density and mixed land use is found as important factors for the choice to walk and cycle (see e.g., [13, 19, 25, 30, 32, 36]). Ding et al. [8] analyse how individuals’ choice to walk and cycle to and from work in China is affected by attitudinal factors, and the results indicate that attitudes to non-motorized travel modes affect the choice.

Studies that focus more directly on walking and walkability often reside in theories for walking behaviour. For example, Alfonzo [1] provides a social-ecological model in combination with a hierarchy of walking needs model, to explain how individual, group, regional and physical-environmental factors affect walking in the behavioural decision-making process. The hierarchy of walking needs moves from feasibility and accessibility in the bottom, towards safety, comfort and pleasurability in the top, illustrating that there are fundamental needs that need to be satisfied before other conditions are considered. Mehta [23] expands this theory by adding a sense of belonging to enhance the walking experience (given a safe and comfortable setting), when performing an empirical survey in the Boston metropolitan area. Yang and Diez-Roux [39] studies how the attitude towards walking and the neighbourhood environment interacts to influence walking behaviour. In an empirical study of six larger cities in the US, they find that the majority of the respondents reported positive attitudes towards walking, and that women, younger respondents, and those with higher income and education were more likely to have a positive attitude towards walking. Perceptions of the neighbourhood environment were also found to affect walking behaviour.

Previous research directed mostly towards cycling also find that the built environment and socio-economic factors influence the choice to cycle. Winters et al. [37] investigates 1902 current and potential cyclists in Metro Vancover and find that the built environment has a significant influence on the choice to cycle (vs. driving), and also that cycling were more common in areas with higher population density. Gender differences in bicycling behaviour is addressed by Krizek et al. [22] when studying surveys of revealed behaviour in Minneapolis. The results show that women cycle less than men, and men are more likely to cycle to work compared to women. Ji et al. [18] confirms this, as women are less likely to use public bicycle to access rail transit in Najing, China. Issues regarding where cyclist prefer to ride, when analysing GPS data, finds that route preferences differ depending on travel purpose, as cyclist travelling to work are more sensitive to distance [5]. In this study we focus on factors that may enhance or prevent the use of active transport modes when commuting, i.e. we focus on walking and cycling jointly.

The vast majority of the research on transport mode is based on data collected in metropolitan areas while knowledge about factors that influence this choice in less populated or rural areas, where public transport generally is less developed, is more limited. This study contributes to this area of research by its explicit focus on the choice of transport mode also outside densely populated areas. Knowledge about important motives for the choice to walk or cycle when commuting, and how these may differ regionally and locally is necessary for policy makers to be able to implement effective policies that also can be accepted by citizens in the entire country, in particular since the scope for effective public transport may be limited in sparsely populated areas.

## Factors affecting the choice to walk or bike

Individuals are assumed to choose to walk or cycle to work or study if it generates the highest expected utility (or the lowest disutility) given its associated costs.^3^ It is anticipated that the choice of transport mode is affected by the monetary and non-monetary costs associated with options available, but also by attitudinal factors such as concerns for health and the climate. Since there is no variable monetary cost associated with walking or cycling, this alternative should be considered attractive for people who prioritise travelling at low expenses.

The characteristics of the available transport modes vary locally, depending on the built environment (such as sidewalks and cycling lanes and access to public transport) and individually (such as distances to work/study). Alternative travel modes are more available in larger cities, while for instance public transport is very limited or absent in the smallest cities. Although walking and cycling is more time consuming, at least for long distances, travel time includes door-to-door and do not vary much over days as traffic congestion or disturbances to public transport is not influencing walking and cycling to the same extent as driving and public transport [14, 26, 29]. Nevertheless, for those with a long distance to work/study, walking and cycling may imply more effort and longer travelling times than what is considered reasonable [16]. Another advantage with walking is that it is accessible for everyone that is able to walk. It is also reasonable to expect that access to relatively safe routes affect the attractiveness of walking or cycling. Differences in the perceived quality of different transport modes can be substantial within as well as between municipalities.

The importance of individual norms and environmental attitudes have also been identified as important factors for the choice of transport mode ([9, 28, 33]; Nyborg et al., [24]) The choice of transport mode is thus not only affected by differences in costs and travel quality, it can also be affected by a strive to reduce the environmental and climate impact from transport. Different citizens will however give different weight to the characteristics of different travel modes, some may consider safety aspects to be more important than travel quality or comfort, while others may consider environmental impacts to be most important. For someone who is concerned with environmental quality and the impacts of climate change, choosing a transport mode that does not generate any greenhouse gases (i.e. walk or cycle) may be considered the best alternative even if it would be more time consuming. We anticipate people who are concerned with environmental quality and climate change to be more likely to walk or cycle than people less concerned about these issues.

Another factor that may affect the choice of transport mode is its expected health effects. Someone with preferences for a healthy lifestyle may prefer to walk or cycle to work or study even if it increases travelling time. Choosing to walk or cycle when commuting may generate increased personal benefits both through reducing future risk of morbidity and mortality and through improved self-image by undertaking a behavior considered morally superior [20, 35]. Improved population health also creates benefits for the society. Finally, the choice of travel mode may also be related to socio-demographic differences such as gender, age, education level and income (see e.g., [2, 8, 13, 18, 39]).

## Survey design, data collection and descriptive results

### The municipalities

Data is collected through a survey sent to households in five selected municipalities in Sweden. The motivations for selecting these municipalities were that they display significant socio-economic, infrastructural and geographical differences. One city is a growing metropolitan municipality (Stockholm), two mid-sized municipalities (Örebro and Luleå), and two small and rural municipalities with static or declining populations and limited access to public transport (Gislaved and Arvidsjaur). The municipalities vary with respect to geographic and climate characteristics as well. Selected statistics related to demographics and transport in each municipality are summarised in Table 1.

**Table 1:** Selected characteristics in the municipalities

	Metropolitan Stockholm	Mid-size Örebro	Mid-size Luleå	Small/rural Gislaved	Small/rural Arvidsjaur
*Population*	960,000	152,000	78,000	30,000	6500
*Municipal center*	1,5 million	115,000	44,000	10,000	4700
*Population change*	Growing	Growing	Slow growth	Static	Shrinking
*Population density*	5197/km^2^	113/km^2^	37/km^2^	25/km^2^	1/km^2^
*Public transport*	Subway, commuter trains, train, bus	Bus, train	Bus, (train)	County bus	County bus
*Kilometers driven/ person/year (average)*	5600 (6733)	6159 (6733)	6922 (6733)	8177 (6733)	8197 (6733)
*Cars/1000 inhabitants (average)*	370 (477)	437 (477)	509 (477)	559 (477)	591 (477)
*Local policies/ measures*	Congestion charge, high parking fees, car pools, environ. Zones	Car pools, parking fees (relative low)	Parking fees	No parking fees	No parking fees
*Household income (yearly in KSEK)*	592,5	425,6	430,1	461,6	373,0
*Income in relation to national average (percent)*	123,7	88,9	89,8	96,4	77,9

Sources: Statistics Sweden, [34] (scb.se)

Stockholm city is the largest city in Sweden. The population is growing and the population density is high. Örebro is the sixth largest municipality in Sweden, it is also growing with a relatively high population density. Both these cities are located in the middle/southern part of Sweden. Luleå is located on the coast in the northern part of Sweden, it is a mid-sized municipality in Sweden with a relatively slow population growth. Gislaved and Arvidsjaur are both small rural municipalities with static or shrinking population and low population densities. The density of the municipality Gislaved is low, although the city is relatively small it is spread out on eight smaller centres. Gislaved is located in the southern part, and Arvidsjaur in the northern part of Sweden.

The population size and density affect the conditions for walking and cycling as distances are on average shorter in smaller and more dense cities. The size and distribution of residential areas impact however also the potential – and cost – for providing effective public transport. The proportion of citizens in each municipality that lives in or near the city center is also relevant, as individuals in the periphery of larger municipalities can have access to similar, more constrained, transport systems as individuals in rural municipalities.

Regarding public transport, there are more alternatives available in larger municipalities, and in particular in Stockholm, compared to the smaller municipalities. In the smallest and rural cities (Gislaved and Arvidsjaur), basically the only alternative to walking or cycling is to go by car, since there are no local buses, only county buses whose route and time table are limited and mainly adjusted to school hours. This is also reflected in the statistics for average number of cars per inhabitant and how many kilometers each person drives per year, which varies between 5600 in Stockholm and 8197 in Arvidsjaur (the national average is 6733 km per inhabitant and year).

Above the national taxes and subsidies on fuels and cars, there are also examples of local transport policies. Most of these aim at reducing car traffic rather than promoting walking and cycling directly. Stockholm has for instance implemented measures such as: a congestion charge, relatively high parking fees, car pools and environmental zones, aimed to reduce local problems with congestion and air pollution. Although all municipalities prioritise walking and cycling lanes when it comes to snow clearance, the smaller rural municipalities (Gislaved and Arvidsjaur) have basically not implemented any additonal local transport policies aimed to reduce car traffic, nor to promote walking and cycling.

There are substantial income differences between households in the municipalities. Households in Stockholm have the highest average income, almost 24% higher than the average household income in Sweden. In the smallest rural city in the north (Arvidsjaur) average household incomes are instead about 22% lower than the national average.

### Survey development and data collection

The first part of the survey collected socio-demographic information. The second part included questions about how the respondents transport themselves to work/study, the distance and frequency of these trips and what factors that are considered important when respondents decide whether to walk or cycle. The development of the questionnaire was supported by a reference group, in which people from the Swedish Environmental Protection Agency and local officials from each municipality participated. The survey was also tested in a small pilot (consisting of about 10 graduate students) and in a pre-test in a later stage with 20 individuals with no specific prior knowledge about environmental impacts from the transport sector or transport policies. As a result of these pre-tests some questions were added, and some questions were rephrased.

The survey used a web panel owned by the company Norstat, collecting at least 200 responses in each municipality in May 2019 (almost a year before the outbreak of the covid-19 pandemic). In the rural and sparsely populated municipalities (Gislaved and Arvidsjaur) the participants in the web panel were not sufficient, responses from these municipalities were therefore supplemented with phone interviews (performed by the same company).

### Descriptive statistics and selected survey responses

In total 1173 individuals responded to the survey, 704 reported that they regularly commute to work/school. Among these 88 respondents had a distance to work/school exceeding 30 km, which is considered too long for walking or cycling (even with electric bikes), and these responses were therefore excluded from the sample. Thus, the final sample consisted of 616 individuals.

Table 2 presents selected sample characteristics in comparison with the population in each municipality (in parentheses) and with the national average (for the pooled sample). Our respondents are older than the average populations from which they were drawn, which is likely, at least to some extent, a result of the survey targeting individuals above 18, while the averages of the populations in parenthesis include also younger individuals. It is still possible that older people are over-represented in the sample. We note that women are overrepresented in all municipalities, and that the average incomes are considerably higher in Stockholm than in other parts of the country, which is also reflected in our sample. Our sample show slightly less variation in income between the municipalities compared to the populations, but the relative order of the averages between municipalities (i.e. lowest income in Arvidsjaur and highest in Stockholm) is reflected. The level of education (measured as percentage with education level above high school) is varying between municipalities, which is also reflected in our sample. The education level in our sample is however higher, both compared to the national average and municipality populations. If the participants in the survey have different characteristics or preferences than the non-participants, this sample selection may lead to biased results. The impact of socio-demographics on the choice to walk/cycle will be examined below.

**Table 2:** Descriptive statistics

	Pooled sample	Metropolitan Stockholm	Mid-size Örebro	Mid-size Luleå	Small/rual Gislaved	Small/rural Arvidsjaur
*Age, years*	46 (41)	44 (39)	46 (40)	44 (42)	48 (42)	48 (46)
*Females, percent*	56 (50)	54 (51)	59 (50)	59 (49)	55 (49)	58 (49)
Household income, kSEK/month^*^	40–60 (40)	40–60 (49)	40–60 (35)	40–60 (36)	40–60 (38)	20–40 (31)
*Higher education, percent*	50 (36)	63 (53)	53 (39)	54 (40)	33 (19)	31 (21)
*Average distance to work, km*	9.0	10.1	8.5	8.2	10.6	6.0
*Often/always walk or bike, percent*	35	32	46	43	23	36

^*^10 kSEK is equivalent to 1 kEuro. Monthly household income is measured in the following categories; 1. < 10, 2. 10–20, 3. 20–40, 4. 40–60, 5. 60–80, 6. 80–100, 7. 100–150, 8. 150–200, 9. > 200. The average of the interval number for the pooled sample and each municipality is 4.2 (pooled), 4.5 (Stockholm), 4.3 (Örebro), 4.1 (Luleå), 4.2 (Gislaved), 3.7 (Arvidsjaur)

**Fig. 1:**
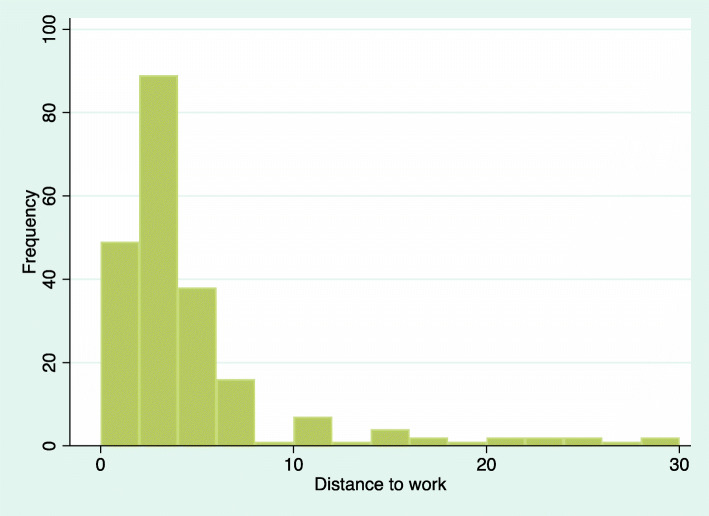
Frequency distribution over distance to work for those that walk/cycle

The last rows in Table 2 present the average distance to work in kilometers in each municipality, and the proportions reporting that they often or always choose to walk or bike when commuting. The average distance to work for those that report that they often/always walk or cycle is 4.4 km, which is considerable shorter compared to the whole sample (9.0 km), indicating as expected that those that have shorter distances more often use active transport modes such as walking or cycling. The frequency distribution regarding distance to work for those that walk/cycle when commuting is presented in Fig. 1, which illustrates that a majority have a relatively short distance to work.^4^ There are only quite few respondents with more than 15 km distance reporting that they often or always walk or cycle. It is likely that these individuals combine walking or cycling with other transports, e.g. public transport. Regarding differences between municipalities it is noted in Table 2 that the average distance to work is the longest in one of the small rual cities (Gislaved) and shortest in the one of the other small rural cities (Arvidsjaur). These differences reflect the different densities of the cities, while Gislaved is spread over several local centers, Arvidsjaur is more concentrated.

In the pooled sample, about 35% report that they often or always walk or cycle to work. This is a relatively high proportion, which to some extent reflect that the survey was conducted in May, which can increase the use of active transport modes as the weather generally is mild. The proportion individuals reporting that they often or always walk or cycle when commuting to work/study also varies across municipalities. It is highest in the two mid-sized cities (Örebro and Luleå), and lowest in the rural and less dense city of (Gislaved) where the share reporting that they often or always walk or bike to work/study is about half compared to in the mid-sized cities (Örebro and Luleå). The proportion in Stockholm corresponds to 32%, which is similar to the proportion in the smallest but relatively concentrated city (Arvidsjaur). Although these differences in average distance between municipalities is interesting and will likely affect the different commuting patterns in municipalities, the econometric analysis will primarily focus on scrutinising the impact of individual differences in distance on walking and cycling.

Table 3 presents survey responses to questions about respondents’ perceptions about the availability and quality of the transport infrastructure (for public transportation, walking/bicycling, and driving) in their municipality. The figures presented are the percentage of the respondents that agree, fully or to a high extent, to each statement (on a five-graded Likert-scale). The responses to the statement about the availability of sidewalks and cycling lanes indicate that respondents in the mid-sized cities (Örebro and Luleå), who also are relatively prone to walk or cycle when they commute, are more satisfied with this infrastructure, compared to respondents in the other municipalities.

**Table 3:** Perceptions about infrastructure in selected municipalities (% agree)

	Metropolitan Stockholm	Mid-size Örebro	Mid-size Luleå	Small/rural Gislaved	Small/rural Arvidsjaur
*Public transport works well*	78	48	50	20	4
*Public transport is expensive*	56	41	52	24	30
*Availability of sidewalks and cycling lanes is high*	68	80	74	57	46
*Availability of parking spaces is high*	12	10	31	70	73
*Traffic congestion occurs frequently*	77	59	13	4	2

The differences across municipalities in opinions about the infrastructure for walking/cycling are however smaller than for infrastructure for public transport and driving cars. When it comes to infrastructure for driving cars, responses differ more substantially, the inhabitants in the smallest rural cities seem to be the most satisfied – while the opposite applies for Stockholm inhabitants. Respondents living in one of the dense mid-sized cities (Örebro) consider traffic congestion to be frequent to a high extent and access to parking as satisfying to an even lower extent than those living in metropolitan Stockholm. Traffic congestion and limited access to parking may, together with access to specific lanes for walking and cycling, contribute to the relatively high proportion walking or cycling in mid-size Örebro, the largest proportion, 80%, agree that infrastructure for active transport are highly available in Örebro.

A large majority, almost 80%, of the respondents in Stockholm agree to the first statement “The public transport works well in my municipality” while only 4% respondents living in small rural Arvidsjaur agree to the same statement. These responses are not surprising, as indicated in Table 1 above, the infrastructure for public transport in Stockholm is superior to the other cities, while access to local public transport is very limited in Gislaved, and non-existent in Arvidsjaur.

The differences in distribution of these responses across municipalities reveal that both the infrastructure for walking and cycling, and access to alternatives to walking or cycling differ subtantially. Access to public transport is highly restricted for people living in the small rural cities (like Arvidsjaur or Gislaved), where car is basically the only alternative to walking or cycling. In Stockholm, on the other hand, public transport is well developed while common problems with parking and congestion instead are related to car use.

## Results – factors affecting the choice to walk or bike

A binary choice logit model is applied to analyse which factors that are related to the choice to often or always choose to commute by walking or cycling (for details see e.g. [15]). The dependent variable is a dummy, equal to one for those who report that they often or always walk or cycle to work/study, and zero otherwise. Table 4 presents variable definitions and coding (see Table 7 in Appendix for correlations between the included variables).

**Table 4:** Variable definitions

*Variable*	Definition	Mean	Std.dev.	Min	Max
*Female*	Dummy variable for gender (1 for female, 0 otherwise)	0.56	0.50	0	1
*Education*	Dummy variable (1 for university degree 0 otherwise)	0.50	0.50	0	1
Income*	Monthly gross income levels for households, ranging between 1 and 9	4.198	1.612	1	9
*Age*	Age ranging from 18 to 82	46	14	18	82
*Distance*	Distance from residence to work or study (kilometers)	9	8	0	30
*Availability WB*	Dummy variable (1 for agree highly or completely to a statement that the availability of sidewalks and biking lanes is high)	0.68	0.47	0	1
*Climate*	Dummy variable (1 for agree highly or completely to a statement that the respondent is concerned about climate change, 0 otherwise)	0.54	0.50	0	1
*Health*	Dummy variable (1 for agree highly or completely to a statement that transport choices are affected by health considerations, 0 otherwise)	0.31	0.46	0	1

*Household income is measured in KSEK (10 KSEK is equivalent to 1 KEuro) in the following categories; 1. < 10, 2. 10–20, 3. 20–40, 4. 40–60, 5. 60–80, 6. 80–100, 7. 100–150, 8. 150–200, 9. > 200

Estimation results for the pooled sample and for each municipality are presented in Table 5. The first column in Table 5 presents results from the estimations based on the pooled sample. As the estimated coefficients can only be interpreted with respect to signs, the negative sign of the female parameter suggest that the probability that a woman will walk and cycle when commuting is lower than the probability that a man will do so. The negative sign of the income parameter indicate that people with higher income are less likely to walk or cycle when commuting than people with lower income (significant at 11% significance level). This is not surprising as walking/cycling is not associated with monetary costs, at least to the extent that driving is. Other socio-economic variables, such as age and education, do however not significantly influence the choice to walk/cycle when commuting in the model based on the full sample.

**Table 5:** Estimation results, binary logit models (standard errors in parentheses)

	Pooled sample	Metropolitan Stockholm	Mid-size Örebro	Mid-size Luleå	Small/rural Gislaved	Small/rural Arvidsjaur
*Constant*	0.432 (0.530)	−2.028 (0.961)**	2.342 (1.440)	3.582 (1.357)***	1.657 (1.388)	−2.353 (1.688)
*Female*	−0.359 (0.215)*	− 0.355 (0.401)	−0.780 (0.591)	− 0.685 (0.576)	−0.112 (0.569)	0.449 (0.613)
*Age*	−0.006 (0.008)	− 0.001 (0.013)	− 0.007 (0.021)	− 0.040 (0.021)*	0.012 (0.021)	0.003 (0.020)
*Education*	0.172 (0.213)	0.285 (0.402)	− 0.209 (0.533)	− 0.333 (0.520)	− 0.606 (0.613)	0.788 (0.621)
*Income*	−0.107 (0.066)	0.181 (0.114)	− 0.324 (0.157)**	− 0.045 (0.175)	− 0.537 (0.254)**	0.101 (0.280)
*Distance*	−0.185 (0.023)***	−0.138 (0.034)***	−0.244 (0.062)***	−0.301 (0.074)***	−0.210 (0.056)***	−0.183 (0.088)**
*Availability WB*	0.478 (0.230)**	1.308 (0.442)***	0.704 (0.773)	−0.114 (0.615)	−0.533 (0.586)	0.999 (0.597)*
*Climate*	0.445 (0.214)**	0.171 (0.434)	0.112 (0.522)	0.515 (0.508)	1.045 (0.592)*	0.848 (0.584)
*Health*	1.210 (0.218)***	1.550 (0.399)***	1.597 (0.599)***	0.997 (0.558)*	0.807 (0.657)	1.322 (0.578)**
*Örebro*	0.508 (0.299)*					
*Luleå*	0.344 (0.293)					
*Gislaved*	−0.341 (0.319)					
*Arvidsjaur*	− 0.338 (0.335)					
*Log- likelihood*	− 297.90	−91.98	−48.78	−52.64	−45.11	−41.08
*Restr. log-likelihood*	−399.69	− 122.32	−73.79	−76.49	−62.94	−55.77
*McFadden Pseud R-sq*	0.255	0.248	0.339	0.344	0.283	0.263
*Number of observations*	616	196	107	112	116	85

*presents significant at 10% significance level, **significant at 5% significance level, ***significant at 1% significance level

Distance to work (in kilometers) is an important factor for the choice to walk/cycle when commuting, as distance increases time costs, and, again the negative sign of the distance parameter, reveal that the likelihood of walking or cycling decreases with distance. If individuals report that they consider availability of sidewalks and cycling lanes to be good in their municipality, they are more likely to choose this transport mode. Attitudinal and norm based factors seem also to be important; respondents that report that they are concerned about climate change and that they consider their health when deciding on transport mode are more prone to walk or cycle when commuting.

To be able to evaluate also the magnitude of the impacts the marginal effects have been calculated (see Table 6 in Appendix). The marginal effects indicate that differences in health considerations are more important for the choice to walk and cycle when commuting, than differences in climate concern and perceptions about the availability of sidewalks and cycling lanes. Hence, although the average respondent reports to be, on average, more concerned for climate change than for his/her personal health, when deciding on transport mode health concern has a larger impact on the choice probability (to choose to walk or cycle) than climate concern. Availability of infrastructure for walking and cycling has however also a relatively substantial impact on the probability of walking or cycling, and it is slightly more important than climate concern.

The municipal dummy variables capture other factors related to municipal differences affecting the choice to walk or cycle than those included as explanatory variables, such as differences in the structure and quality of the built environment and infrastructure. Metropolitan Stockholm is used as reference category. The positive sign of the parameter estimates for the mid-size citiy Örebro indicate that walking and cycling is more common in this municipality, compared to Stockholm. As the coefficients for the other cities (Luleå, Gislaved and Arvidsjaur) are not statistically significant we cannot conclude that walking or cycling is more or less common in these municipalities compared to Stockholm.

The results from estimating the model for the individual municipalities reveal that somewhat different factors seem to be important in different types of municipalities. Regarding the socio-economic variables we note that in both mid-sized Örebro and small rural Gislaved it is more common that those with a lower income more often choose to walk or cycle when commuting. In the mid-size city Luleå, it is more common that younger people choose to walk or cycle. Distance to work/school is an important factor for all municipalities, respondents with long distance are less likely to commute by walking or cycling. Regarding availability of sidewalks and cycling lanes, results indicate that both in metropolitan Stockholm and the smallest and rural city Arvidsjaur it is more common that those that agree to the statement that the availability of sidewalks and cycling lanes is good more frequently chooses to walk or cycle. Climate concern seem to motivate commuters to walk or cycle in Gislaved, but not in the rest of the municipalities. Health considerations are important for the residents that choose to walk/cycle in all municipalities, except in Gislaved. The marginal effects for the separate municipalities (see Table 6 in Appendix) are similar to those estimated for the pooled sample, indicating that health considerations, in comparison, are relatively important for the choice to walk/cycle when commuting.

## Discussion and concluding remarks

The main purpose of this paper was to analyse individual motives for the choice to walk/cycle when commuting in different local contexts, using different parts of Sweden as our example. Results are based on 616 survey responses from five different municipalities. The results indicate that the differences in the extent to which inhabitants walk or cycle to and from work are related to distance, concerns for the environment, personal health considerations, and to perceptions about local differences in the built environment (the availability of sidewalks and cycling lanes). Respondents considering walking and cycling lanes to be highly accessible are more likely to walk or cycle to work than respondents who consider this infrastructure to be lacking. This is consistent with the findings of previous research, showing that the characteristics of the built environment have a significant impact on choice of non-motorised travel modes (see e.g. [6, 12, 37]).

We do not find conclusive support for the idea that people using active transport modes are those that have less access to car, like younger citizens and those from lower income groups, as the impact of demographic and socio-economic characteristics are mixed. The results on the pooled sample suggest that women are less prone to walk or cycle when commuting than men, which is consistent with the findings of Khan et al. [19], Krizek et al. [22] and Vandenbulcke et al. [36]. Previous research stress that one explanation for this result is that women often travel in more complex ways, due to family commitments (Janet et al., [17]). Khan et al. reported household income to be positively related to the probability of using non-motorized travel modes, while in this study we found, similar to Vandenbulcke et al. [36], that household income is negatively related to walking or cycling. In one of the rural city (Gislaved) access to public transport is very limited, which may help explain why people with lower income in this city are more prone to use active transportation, than people with higher incomes (that more often have a car available). This indicates that people with few alternatives available, are more likely to walk or cycle.

The alternatives available to walking or cycling differ between the municipalities. Higher proportions of respondents in the mid-sized cities (Örebro and to some extent Luleå) report that they commute by walking or cycling than in the other municipalities. A plausible explanation for respondents in metropolitan Stockholm being less prone to to walk or cycle can be that they have access to well developed public transport, while in the small rural municipalities (Gislaved and Arvidsjaur) public transport is more or less absent, while access to parking is readily available at low cost and there is no congestion so car is a more attractive alternative than in metropolitan Stockholm.

In addition, in more sparsely populated areas, it is challenging to provide well-functioning public transport at reasonable cost. Therefore, the alternatives available for more sustainable transport outside metropolitan areas and large cities are often restricted to either fossil-free cars or non-motorised transport modes such as walking and cycling. While public transport is costly from a societal perspective, electric vehicles are still expensive and thus limited with regard to accessibility from an individual perspective. Increased walking and cycling is however accessible in all parts of the country, and could also potentially generate health benefits for both individuals and society.

With regard to personal attitudes, we find that concern both for the environment and for personal health are positively related to walking and cycling, and it is potentially important to stress these benefits when polices and campaigns aiming for a more sustainable transport sector are designed. In particular health considerations have a relatively large impact on the probability that a respondent report to be walking or cycling to work often or always, and we believe that health motives are somewhat under-utilised when sustainable transport are promoted. Not least considering that the ongoing pandemic may have increased interest in issues important for personal health, and at the same time made the alternatives accessible for sustainable transport more restrictive, since the general public is advised not to use public transport. This may constitute a window of opportunity for increasing the use non-motorised travel modes such as walking and cycling. To achieve this, it is however important to consider availability and accessability aspects in community planning, and to prioritise safety and comfort of walking and cycling, not least in parts of the country where public transport is not an economically viable option.

In this study we have treated pedestrians and cyclists as a group of active travellers, however our results indicate that pedestrians and cyclists is a heterogeneous group and the motives for walking or cycling may differ. We thus agree with [29], p.136) that it is an interesting area for future research to study walkers and cyclists separately. This would however require other data than collected in this study, for example, about individual attitudes towards sports and recreation.

One limitation of this study is the sample sizes, not least in Stockholm where the sample constitute a very small fraction of the population. Also the number of municipalities is limited. Nevertheless, although the results can of course not be generalised to all small or rural cities, as there are likely specific circumstances in each municipality that need to be addressed, many small and rural cities share similar characteristics. For example, many face constrained resouces, limited and perhaps shrinking populations, which implies that developing well functioning public transport is out of reach for many of these municipalities. To walk or cycle can thus be seen as an accessible alternative that is available also outside densely populated areas. The results presented point at the importance of considering the local context – also outside metropolitan areas – when local transport polices are designed. We therefore recommend decision makers on the local level to use planning instrument to improve accessibility, safety and comfort for those using active transport modes.

Although there are indications of sample selection in the sense that women and highly educated are over represented, we found no or very limited evidence that gender or educational level affects the choice to walk or bike when commuting, and therefore selection biases stemming from this are likely not severe. As well, given that health considerations are important for the choice to walk or bike when commuting it would be interesting to analyse these in more detail; and for instance scrutinise the importance of differences in attitudes towards physical activity in itself, in relation to transport choice. We had however not access to any such data, but this is an area for future research.

## Data Availability

The data which the analysis is based on will be made available upon request.

## Appendix

**Table 6:** Estimated marginal effects (standard errors in parentheses)

Average marginal effects *Pr (walkbike), predict dy/dx w.r.t.*	Pooled sample	Metropolitan Stockholm	Mid-size Örebro	Mid-size Luleå	Small/rural Gislaved	Small/rural Arvidsjaur
*Female*	−0.058 (0.035)*	−0.055 (0.062)	− 0.118 (0.087)	− 0.102 (0.084)	− 0.014 (0.073)	0.073 (0.098)
*Age*	−0.001 (0.001)	0.000 (0.002)	− 0.001 (0.003)	− 0.006 (0.003)*	0.002 (0.003)	0.001 (0.003)
*Education*	0.028 (0.035)	0.044 (0.062)	0.032 (0.081)	−0.050 (0.077)	−0.077 (0.077)	0.127 (0.096)
*Income*	−0.017 (0.011)	0.028 (0.017)	− 0.049 (0.157)**	−0.007 (0.026)	−0.069 (0.030)**	0.016 (0.045)
*Distance*	−0.030 (0.003)***	−0.021 (0.005)***	−0.037 (0.006)***	−0.045 (0.008)***	−0.027 (0.005)***	−0.030 (0.013)**
*Infrastructure WB*	0.078 (0.037)**	0.203 (0.063)***	0.107 (0.116)	−0.017 (0.092)	− 0.068 (0.074)	0.161 (0.090)*
*Climate*	0.072 (0.034)**	0.027 (0.067)	0.017 (0.079)	0.077 (0.074)	0.133 (0.071)*	0.137 (0.090)
*Health*	0.197 (0.032)***	0.240 (0.052)***	0.242 (0.079)***	0.149 (0.078)*	0.103 (0.081)	0.213 (0.081)***
*Örebro*	0.082 (0.048)*					
*Luleå*	0.056 (0.047)					
*Gislaved*	−0.055 (0.052)					
*Arvidsjaur*	−0.055 (0.054)					
*Number of observations*	616	196	107	112	116	85

*presents significant at 10% significance level, **significant at 5% significance level, ***significant at 1% significance level

**Table 7:** Correlation coefficients

	WB	Female	Age	Edu	Income	DW	WB_Avail	Climate	Health	Örebro	Luleå	Gislaved	Arvidsjaur
Walk_Bike	1000												
Female	0,019	1000											
Age	−0,042	−0,127	1000										
Education	0,060	0,099	−0,091	1000									
Income	−0,061	0,009	0,054	0,119	1000								
Dist_Work	−0,412	−0,050	0,036	0,011	0,060	1000							
WB_Avail	0,170	−0,037	0,054	0,067	−0,020	− 0,139	1000						
Climate	0,119	0,214	−0,055	0,144	0,074	−0,021	0,010	1000					
Health	0,298	0,132	0,057	0,090	0,003	−0,135	0,108	0,222	1000				
Örebro	0,101	0,024	0,006	0,032	0,026	−0,026	0,135	−0,004	0,030	1000			
Luleå	0,075	0,025	−0,051	0,044	−0,037	− 0,045	0,057	− 0,002	0,033	− 0,216	1000		
Gislaved	−0,121	−0,011	0,071	−0,165	− 0,010	0,094	− 0,101	−0,011	− 0,095	−0,221	− 0,227	1000	
Arvidsjaur	0,010	0,011	0,078	−0,154	−0,131	− 0,148	−0,085	− 0,016	−0,011	− 0,183	−0,189	− 0,193	1000

## Footnotes

1 Although walking and cycling have been favourable from a contamination perspective during the covid-19 pandemic, the permanent effects of the pandemic on active transport modes are yet not known.

2 The following municipalities are selected (in declining population sizes): Stockholm (more than 900,000 inhabitants), Örebro, Luleå, Gislaved and Arvidsjaur (with 6300 inhabitants).

3 Although we apply a rationalist approach, we acknowledge that it is possible that transport choices may, to some extent, be affected by bounded rationality. It is however beyond the scope of this study to analyse such effects.

4 A higher proportion of walkers compared to cyclists have shorter distance to work (as expected). Also, the average distance to work for those reporting that they walk is 3.9 km, to be compared with 4.7 km for those that report that they cycle.
